# Evaluation of anti-quorum sensing and antibiofilm effects of secondary metabolites from *Gambeya lacourtiana* (De Wild) Aubr. & Pellegr against selected pathogens

**DOI:** 10.1186/s12906-023-04115-4

**Published:** 2023-08-24

**Authors:** Rostan Mangoua Talla, Alfred Ngenge Tamfu, Brussine Nadège Kweka Wakeu, Ozgur Ceylan, Céline Djama Mbazoa, Gilbert Deccaux Wabo Fotso Kapche, Bruno Ndjakou Lenta, Norbert Sewald, Jean Wandji

**Affiliations:** 1 Department of Organic Chemistry, Faculty of Science, The University of Yaoundé 1, P.O. Box 812, Yaoundé, Cameroon; 2 Department of Chemistry, Higher Teacher Training C ollege, The University of Yaoundé 1, P.O. Box 47, Yaoundé, Cameroon; 3 Department of Chemical Engineering, School of Chemical Engineering and Mineral Industries, University of Ngaoundéré, P.O. Box 454, Ngaoundéré, Cameroon; 4 Food Quality Control and Analysis Program, Ula Ali Kocman Vocational School, Mugla Sitki Koc-man University, Mugla, 48147 Turkey; 5 Chemistry Department, Organic and Bioorganic Chemistry, Bielefeld University, P.O. Box 100131, 33501 Bielefeld, Germany

**Keywords:** *G. lacourtiana*, Secondary metabolites, Antimicrobials, Quorum-sensing inhibition, Antibiofilm activity, Swarming motility inhibition

## Abstract

**Background:**

Microbial infections cause serious health problems especially with the rising antibiotic resistance which accounts for about 700,000 human deaths annually. Antibiotics which target bacterial death encounter microbial resistance with time, hence, there is an urgent need for the search of antimicrobial substances which target disruption of virulence factors such as biofilm and quorum sensing (QS) with selective pressure on the pathogens so as to avoid resistance.

**Methods:**

Natural products are suitable leads for antimicrobial drugs that can inhibit bacterial biofilms and QS. Twenty compounds isolated from the medicinal plant *Gambeya lacourtiana* were evaluated for their antibiofilm and anti-quorum sensing effects against selected pathogenic bacteria.

**Results:**

Most of the compounds inhibited violacein production in *Chromobacterium violaceum* CV12472 and the most active compound, Epicatechin had 100% inhibition at MIC (Minimal Inhibitory Concentration) and was the only compound to inhibit violacein production at MIC/8 with percentage inhibition of 17.2 ± 0.9%. Since the bacteria *C. violaceum* produces violacein while growing, the inhibition of the production of this pigment reflects the inhibition of signal production. Equally, some compounds inhibited violacein production by *C. violaceum* CV026 in the midst of an externally supplied acylhomoserine lactone, indicating that they disrupted signal molecule reception. Most of the compounds exhibited biofilm inhibition on *Staphyloccocus aureus, Escherichia coli* and *Candida albicans* and it was observed that the Gram-positive bacteria biofilm was most susceptible. The triterpenoids bearing carboxylic acid group, the ceramide and epicatechin were the most active compounds compared to others.

**Conclusion:**

Since some of the compounds disrupted QS mediated processes in bacteria, it indicates that this plant is a source of antibiotics drugs that can reduce microbial resistance.

## Introduction

Bacterial infections lead to various diseases due to the development of pathogenic bacteria in humans or animals. They seriously threaten public health, with an estimated death of over 10 million people per year by 2050 [1]. With time, viruses, bacteria and fungi become resistant to the therapeutic effects of the drugs that they were previously susceptible to [2]. This effect is referred to as antimicrobial resistance and the statistics show that an estimated annual 700,000 human deaths occur as a result of antibiotic resistance [3]. Inappropriate and misuse of antimicrobials contribute to the emergence of resistance in bacteria and it is worse in developing countries since patients can access antibiotics without prescription [4]. Antibiotics which target the inhibition and death of bacterial and fungal cells are falling out of use since they are the faced with resistance. Targeting microbial cell-to-cell communication systems (quorum-sensing) provides a possible solution to overcome this phenomenon. QS mediates the generation, diffusion and reception of small signal molecules that trigger virulence, resistance genes, toxin production, non-tolerance to antibiotics, drug efflux pumps and extracellular polysaccharide synthesis which constitutes the biofilm barrier [5, 6]. Investigating the QS effects of phytochemicals during can shape the development of drugs that target QS-signal and receptors which contributes to antibiotic resistance, motility and biofilm formation [7, 8]. Biofilm must be considered synonymously with antibiotic resistance because of its proficiency in transferring resistance genes between bacterial species and colonies as well as its impermeability and insusceptibility to antibiotics as well as efflux pump systemic elimination of antibiotics [9]. Antibiofilm and quorum-sensing (QS) inhibition are new methods currently employed as suitable strategies to combat microbial resistance and reduce severity of infections [10–12]. For this reason, most researchers are currently engaged in investigating plant products in the search of new therapeutic antibiotic agents that are capable of inhibiting QS processes and control infections without promoting the development of resistant microbial strains [13, 14]. Various medicinal plants are used to treat infectious diseases especially in low-income countries with efficacy. Phytoconstituents such as saponins, alkaloids, flavonoids, terpenoids and tannins have various bioactive functions including antibacterial activity with different mechanisms of action and less chances for microbial resistance [15].

*Gambeya* genus (Sapotaceae) is a pantropical genus of about 80 species widely distributed in West and Central Africa. They are used in folk medicine to treat sterility and various diseases including wounds and vaginal infections [16–18]. Some species such as *Gambeya africana* and *Gambeya boukokoensis* are reported to possess antitumor, anti-inflammatory, antibacterial, antinociceptive, antioxidant, antiplasmodial, antiplatelet, hypoglycemic, hypolipidemic and hepatoprotective activities [16–18]. Phytochemical investigation of some *Gambeya* species from Africa have led to the isolation of diverse classes of compounds, including phytosteroids, saponins, pentacyclic triterpenoids, flavonoids, alkaloids and bi-flavonoids [16–18]. *Gambeya lacourtiana* (De Wild) Aubr. & Pellegr is mostly distributed from Cameroon to the Central African Republic, Gabon and Democratic Republic of Congo. Its vernacular names include ‘abam’, ‘longhi’, ‘longhi rouge’ (French) [17]. In Cameroon, the population in the upper Nyong Valley use the stem bark and leaves of *Gambeya lacourtiana* to treat male sexual impotence and wound infections [17]. It is also administered orally for the treatment of uterine heamorrhage, chlamydia and other vaginal infections. The phytochemical investigation of the fruits of *G. lacourtiana* led to the isolation and characterization of pentacyclic triterpenoids, phytosteroids, ceramide, cerebroside, glycolipid, chlorophyll and carbohydrate [16, 18]. The fact this plant is popularly used in treating wounds, chlamydia and vaginal infections suggests that it possesses antimicrobial activities.

In our continuous search of bioactive natural products, with new modes of action that can reduce the chances of antibiotic resistance, the chemical constituents of *G*. *lacourtiana* were evaluated for their anti-quorum sensing, antimicrobial and anti-biofilm activities. These assays involving the inhibition of virulence factors in pathogenic bacteria were conducted at low concentrations, usually below minimal inhibitions.

## Materials and methods

### Extraction and isolation of chemical compounds

**Fig. 1:**
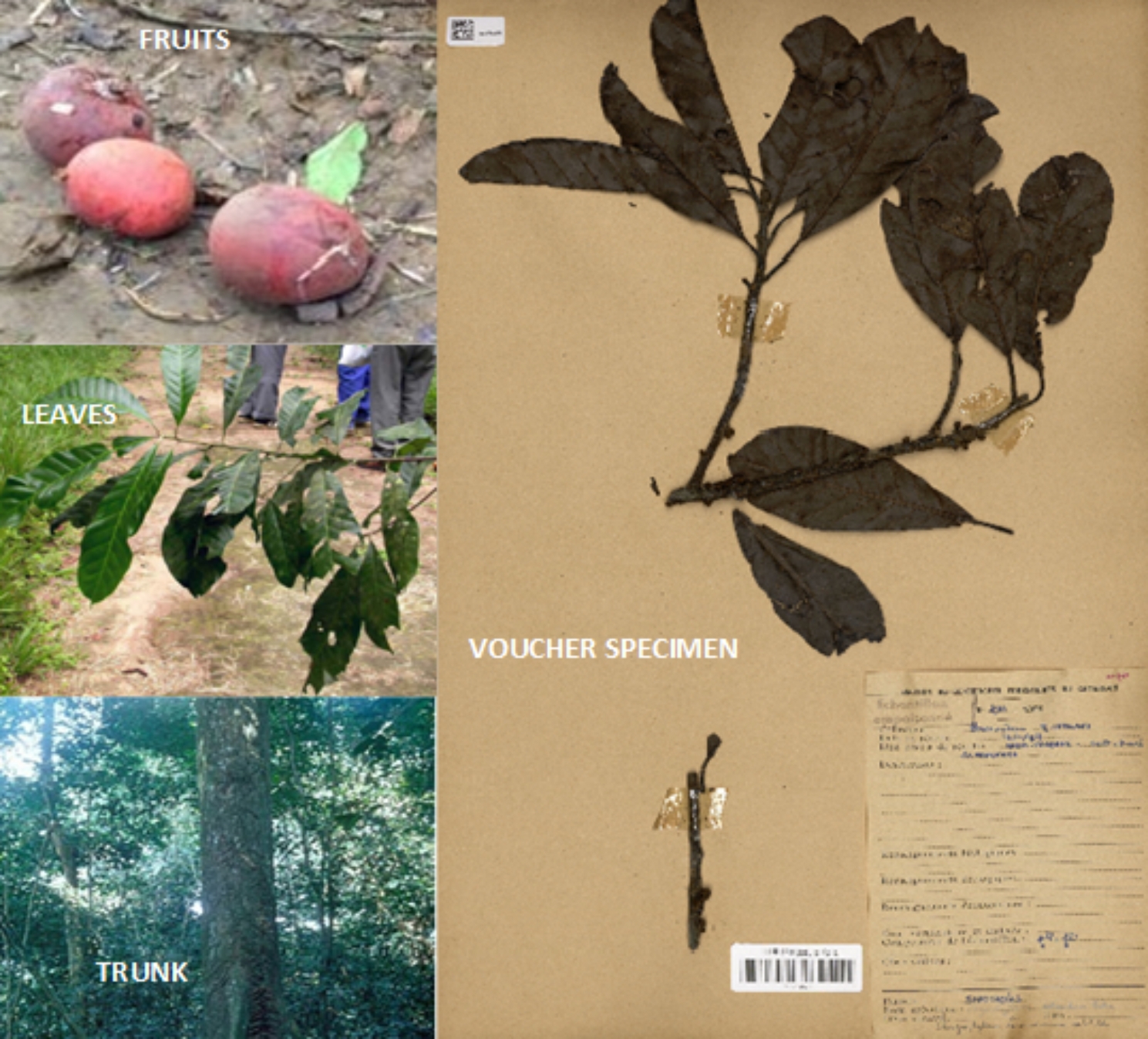
Photo of plant material and voucher specimen of *G. lacourtiana*

**Fig. 2:**
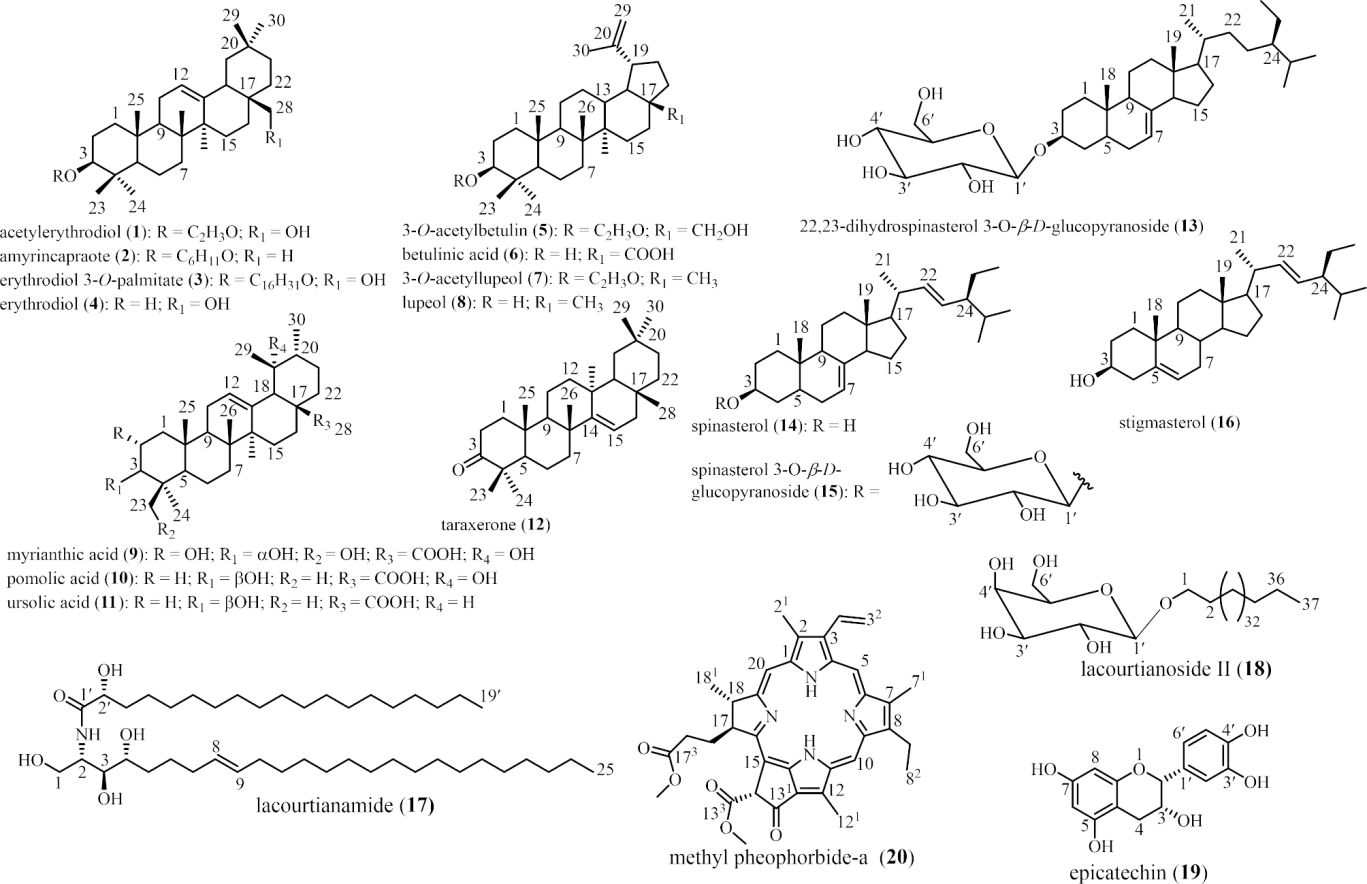
Structures of test compounds isolated from *G. lacourtiana*

The fruits, leaves and stem bark of *G. lacourtiana* were collected in Mbalmayo (Latitude 3°23’ 0.01” North, Longitude 11°36’ 0.00” East) in the Centre Region of Cameroon, in June 2017 and identified by Mr. Victor Nana a botanist at the National Herbarium of Cameroon, where a voucher specimen is deposited under the reference YA0011679. The plant parts studied as well as the voucher specimen are provided on Fig. 1. The plant materials were extracted by maceration and purified using chromatographic methods to yield the compounds which were characterized using ^1^ H NMR and ^13^ C NMR and their structures are given in Fig. 2.

The fruits were chopped into pieces, dried and powdered to obtain 1428.6 g which was extracted by maceration with 5.0 L of methanol for 72 h at room temperature. The solvent was evaporated under vacuum to afford 109.4 g of methanol crude extract. Successive solvent-solvent extraction of the crude extract using *n*-hexane, methylene chloride and *n*-butanol afforded 63.7 g, 6.4 and 9.0 g respectively. A portion of (60.0 g) of the *n*-hexane extract was subjected to column chromatography (CC) over silica gel and eluted with a mixture of *n*-hexane-CH_2_Cl_2_ (0-100%) followed by CH_2_Cl_2_-MeOH (0-100%) to give 100 fractions of 125 mL each which were pooled on the basis of TLC into six sub-fractions (F1 to F6). Sub-fraction F1 (9.2 g), precipitated as a white powder which was further rechromatographed over silica gel with an isocratic system (*n*-hexane/CH_2_Cl_2_ 9/1, v/v) and afforded **Amyrincapraote** (12.1 mg). Sub-fraction F2 (2.0 g) purified on silica gel column with isocratic system (*n*-hexane/CH_2_Cl_2_ 1/4, v/v), yielded **Acetylerythrodiol** (11.3 mg) and **Erythrodiol 3-O-palmitate** (13.5 mg) while sub-fraction F3 (6.0 g) afforded **3-O-acetylbetulin** (15.0 mg) and **Acetylerythrodiol** (20.0 mg) over silica gel using the same eluent phase. Purification of sub-fraction F6 (7.2 g), over silica gel column eluted with a mixture of CH_2_Cl_2_-MeOH (0–25%) afforded **Lacourtianamide** (9.5 mg) and **Lacourtianoside II** (25.2 mg).

The 1038.4 g of *G. lacourtiana* powdered leaves was extracted at room temperature in 10.0 L of a mixture of CH_2_Cl_2_:MeOH (1:1) to afford 112.9 g of crude extract. The crude extract was further extracted successively with *n*-hexane, ethyl acetate and *n*-butanol and 35.0 g, 5.8 and 8.6 g respectively of extracts were obtained. 30.0 g of the hexane extract was subjected to a column chromatography (CC) over silica gel and eluted with a mixture of *n*-hexane-CH_2_Cl_2_ and CH_2_Cl_2_-MeOH gradients to give 120 fractions of 125 mL each which were combined according to their TLC profiles into four sub-fractions (F1 to F4). Sub-fraction F1 (2.2 g), eluted with (*n*-hexane/CH_2_Cl_2_ 90/10 to 80/20, v/v) precipitated few hours later at the ambient temperature, giving **3-O-acetyllupeol** (100.0 mg). Sub-fraction 2 (3.3 g) was subjected to further CC using an isocratic system (*n*-hexane/CH_2_Cl_2_ 4/1, v/v) to afford **Lupeol** (25.0 mg) and **Stigmasterol** (8.0 mg). Sub-fraction 3 (1.5 g) precipitated as a white powder which was filtered and washed with *n*-hexane/EtOAc 4/1 to yield **Erythrodiol** (19.8 mg). A portion of 5.0 g of ethyl acetate extract was subjected to column chromatography (CC) over silica gel and eluted with a mixture of *n*-hexane-ethyl acetate gradients polarity. A total of 90 fractions of 125 mL each were collected and combined according to TLC profile monitoring to two sub-fractions (F1 to F2). Sub-fraction F2 (0.9 g) precipitated as a bluish powder and was filtered and washed with *n*-hexane to afford **Methyl pheophorbide-a** (10.0 mg). A portion of *n*-butanol (8.0 g) was subjected to CC over silica gel and eluted with a mixture of EtOAc-MeOH (0-100%). 50 fractions of 125 mL each were collected and regrouped based on TLC into four sub-fractions (F1 to F3). Successive CC on F1 (3.2 g) with ethyl acetate-methanol (0–20%) led to the isolation of **Epicatechin** (23.0 mg) and **22,23-Dihydrospinasterol 3-O-β-D-glucopyranoside** (18.8 mg).

2507.2 g of powdered stem barks were extracted thrice by maceration, using 8.0 L of methanol for 72 h at room temperature to give 235.8 g of MeOH extract. The crude extract was further extracted successively with *n*-hexane, ethyl acetate and *n*-butanol giving 41.0 g, 5.0 and 50.4 g of extracts respectively. A portion of the *n*-hexane extract (40.0 g) was subjected to flash chromatography (FC) over silica gel while eluting with a mixture of *n*-hexane-CH_2_Cl_2_ (0-100%) followed by CH_2_Cl_2_-MeOH (0–50%). A total 12 fractions of 500 mL each were collected and combined according to TLC profile into four sub-fractions (F1 to F4). Sub-fraction F1 (3.3 g) obtained by FC (*n*-hexane/CH_2_Cl_2_ 25/75; v/v) was further subjected to silica gel column chromatography (CC) with isocratic system (*n*-hexane/CH_2_Cl_2_ 17/3) to yield **Taraxerone** (9.2 mg). Sub-fraction F3 (0.9 g) obtained by FC (*n*-hexane/CH_2_Cl_2_ 25/75; v/v) precipitated and was filtered to afford **Spinasterol** (12.9 mg). A portion of the ethyl acetate extract (4.8 g) was chromatographed over silica gel and eluted with a mixture *n*-hexane-ethyl acetate (0-100%) and ethyl acetate-MeOH (0–50%) gradient. A total of 60 fractions of 125 mL each were collected and combined according to TLC profile monitoring to two sub-fractions (F1 to F3). F3 fraction precipitated in the form of a white solid after purification on CC using *n*-hexane/EtOAc (1/9, v/v) to afford **6** (15.1 mg). Successive CC on F1 (0.6 g) using a mixture of *n*-hexane/EtOAc (0–20%) gradient yielded, **Myrianthic acid** (10.0 mg), **Pomolic acid** (9.1 mg) and **Ursolic acid** (12.7 mg). The sub-fractions F2 precipitated on standing and yielded **Spinasterol 3-O-β-D-glucopyranoside** (8.0 mg).

### Microbial strains and test compounds solution preparations

The microorganisms used in this study were *Staphylococcus aureus* ATCC 25,923, *Escherichia coli* ATCC 25,922 and *Candida albicans* ATCC 10,239 for antimicrobial and antibiofilm assays. *Chromobacterium violaceum* CV12472 and *Chromobacterium violaceum* CV026 were used in the violacein and quorum sensing inhibitions respectively. *Pseudomonas aeruginosa* PA01 was used in the anti-swarming motility assay.

The stock solution of each test compound at a concentration of 10 mg/mL was prepared by dissolving the compounds in H_2_O:DMSO (95:5%, v:v) and serial dilutions were made from each stock solution using distilled H_2_O to further minimize the DMSO used in the stock solution.

### Determination of minimum inhibitory concentrations

Minimal inhibitory concentration (MIC) of each compound was determined by the broth dilution method described previously [19, 20]. The MIC is the lowest extract concentration that yielded no visible microbial growth. The test medium was Mueller-Hinton broth and the density of bacteria was 5 × 10^5^ colony-forming units (CFU)/mL. Cell suspensions (100 µL) were inoculated into the wells of 96-well microtitre plates in the presence of compounds with different final concentrations (1, 0.5, 0.25, 0.125, 0.0625, 0.03125 mg/mL). The inoculated microplates were incubated at 37ºC for 24 h before being read.

### Inhibitory effect of compounds on microbial biofilm formation

The ability of the compounds at MIC and sub-MIC concentrations including 1, ½, ¼, and 1/8 MIC to inhibit biofilm by test microorganisms were evaluated with a microplate biofilm assay [21, 22]. Briefly, 1% of overnight cultures of isolates were added into 200 µL of fresh Tryptose-Soy Broth (TSB) supplemented with 0.25% glucose and cultivated in the presence and absence of compounds without agitation for 48 h at 37 ºC. The wells containing TSB + cells only served as control. After incubation, remove planktonic bacteria were removed by gently washing with distilled water. The biofilms were subsequently stained by filling wells with 200 µL of 0.1% crystal violet solution and then allowed for 10 min at room temperature. Wells were rinsed once more with distilled water using micro-pipette to remove the unabsorbed crystal violet. A volume of 200 µL of 33% glacial acetic acid (for Gram-positive bacteria) or ethanol 70% (for Gram-negative bacteria or fungi) were filled into the wells. After shaking 125 µL was pipetted from each of the wells into a sterile tube and volume was adjusted to 1 mL using distilled water. Finally, optical density (OD) of each well was measured at 550 nm (Thermo Scientific Multiskan FC, Vantaa, Finland). Percentage of inhibition of biofilm by the tested extracts was calculated using the formula:

$$\begin{array}{l}{\rm{Biofilm}}\,{\rm{inhibition}}\,\left( \% \right)\, = \\\,\frac{{OD{{550}_{Control}} - OD{{550}_{Sample}}}}{{OD{{550}_{Control}}}}\, \times \,100\end{array}$$

### Bioassay for quorum-sensing inhibition (QSI) activity using C. violaceum CV026

Inhibition of quorum sensing was determined as described elsewhere [23, 24] with little modifications. 5 mL of lukewarm molten Soft Top Agar (1.3 g agar, 2.0 g tryptone, 1.0 g sodium chloride, 200 mL deionized water) were seeded with 100 µL of an overnight culture of CV026, and 20 µL of 100 µg/mL acylhomoserine lactone (C_6_HSL) was added as exogenous hormone source. This was mixed gently and poured carefully over the surface of sterile solidified LBA plate as an overlay. 5 mm diameter wells were made on each plate after solidification of the overlay and each of the wells were filled with 50 µL of MIC and sub-MIC concentrations of filter sterilized compounds. Each experiment was done in triplicate and the plates were incubated in upright position at 30 °C for 3 days after which the diameters of the quorum sensing inhibition zones were measured. A white or cream-colored halo around this well against a purple lawn of activated CV026 bacteria was an indication of QSI and its diameter was measured in millimeters.

### Violacein inhibition assay using C. violaceum CV12472

The compounds were subjected to qualitative analysis of QSI potentials for their ability to inhibit violacein production by *C. violaceum* ATCC 12,472 [13]. Overnight cultures (10 µL) of *C. violaceum* (adjusted to 0.4 OD at 600 nm) were added into sterile microtiter plates containing 200 µL of Luria-Bertani (LB) broth and incubated in the presence and absence of MIC and sub-MICs of extracts. LB broth containing *C. violaceum* ATCC 12,472 was used as a positive control. These plates were incubated at 30 °C for 24 h and observed for the reduction in violacein pigment production. The absorbance was read at 585 nm. The percentage of violacein inhibition was calculated by following the formula:

$$\begin{array}{l}{\rm{Violacein}}\,{\rm{inhibition}}\,\left( \% \right)\, = \,\\\frac{{OD{{585}_{Control}} - OD{{585}_{Sample}}}}{{OD{{585}_{Control}}}}\, \times \,100\end{array}$$

### Swarming motility inhibition on Pseudomonas aeruginosa PA01

Swarming motility inhibition was evaluated according to a method previously described [25, 26]. Summarily, overnight cultures of *P. aeruginosa* PA01 strain were point inoculated at the center of swarming plates consisting of 1% peptone, 0.5% NaCl, 0.5% agar and 0.5% of filter-sterilized D-glucose with various concentrations of compounds (100, 75 and 50 µg/mL) and the plate without the compounds was maintained as control. Plates were incubated at an appropriate temperature in an upright position for 18 h. The swarming migration was recorded by following swarm fronts of the bacterial cells.

### Statistical analyses

Descriptive statistics were applied on the data obtained. Each experiment was done in triplicate and the means of three parallel measurements were deduced. The values given are means ± SEM (Standard error of the mean) for three measurements. One-way ANOVA (analysis of variance) was used to compare differences amongst the means and were considered statistically significant *p* < 0.05.

### Characteristics of isolated compounds

**Acetylerythrodiol (1)**: White powder; m.p. 237–239 °C; ^1^ H NMR (CDCl_3_, 500 MHz): *δ*_H_ 0.82 (s, Me-24), 0.92 (s, Me-23), 0.95 (s, Me-25), 0.96 (s, Me-26), 0.97 (s, Me-29), 1.06 (s, Me-27), 1.12 (s, Me-30), 1.91 (1 H, dd, *J* = 13.5 and 4.3 Hz, H-18), 3.15 (1 H, d, *J* = 10.8 Hz, H-28a), 3.48 (1 H, d, *J* = 10.8 Hz, H-28b), 4.43 (1 H, m, H-3), 5.12 (1 H, t, *J* = 3.6 Hz, H-12), 1.98 (s, Me-2′); ^13^ C NMR (CDCl_3_, 125 MHz): *δ*_C_ 38.3 (C-1), 23.6 (C-2), 80.9 (C-3), 37.7 (C-4), 55.2 (C-5), 18.2 (C-6), 32.5 (C-7), 39.8 (C-8), 47.5 (C-9), 36.8 (C-10), 23.5 (C-11), 122.3 (C-12), 144.2 (C-13), 41.7 (C-14), 25.5 (C-15), 22.0 (C-16), 36.9 (C-17), 42.3 (C-18), 46.4 (C-19), 30.9 (C-20), 34.1 (C-21), 31.0 (C-22), 28.0 (C-23), 16.7 (C-24), 15.6 (C-25), 16.7 (C-26), 25.9 (C-27), 69.7 (C-28), 33.2 (C-29), 23.6 (C-30), 171.1 (C-1′), 21.3 (C-2′).

**Amyrincapraote (2)**: White powder; ^1^ H NMR (CDCl_3_, 500 MHz): *δ*_H_ 0.76 (s, Me-24), 0.80 (s, Me-23), 0.83 (s, Me-30), 0.89 (s, Me-29), 0.90 (s, Me-26), 1.06 (s, Me-25), 1.25 (s, Me-27), 1.94 (1 H, dd, *J* = 13.5 and 4.3 Hz, H-18), 4.43 (1 H, m, H-3), 5.11 (1 H, t, *J* = 3.3 Hz, H-12), 0.82 (t, *J* = 3.5 Me-6′), 2.22 (2 H, t, *J* = 7.4 Hz, H-2′); ^13^ C NMR (CDCl_3_, 125 MHz): *δ*_C_ 38.3 (C-1), 23.6 (C-2), 80.6 (C-3), 37.8 (C-4), 55.3 (C-5), 18.3 (C-6), 32.6 (C-7), 39.8 (C-8), 47.6 (C-9), 36.8 (C-10), 23.5 (C-11), 121.7 (C-12), 145.2 (C-13), 41.7 (C-14), 26.1 (C-15), 26.9 (C-16), 32.5 (C-17), 47.2 (C-18), 46.8 (C-19), 31.1 (C-20), 34.7 (C-21), 37.2 (C-22), 28.1 (C-23), 16.8 (C-24), 15.6 (C-25), 16.8 (C-26), 25.9 (C-27), 28.4 (C-28), 33.4 (C-29), 23.7 (C-30), 173.7 (C-1′), 34.8 (C-2′), 24.9 (C-3′), 31.4 (C-4′), 22.3 (C-5′), 13.9 (C-6′).

**Erythrodiol 3-*O*-palmitate (3)**: White powder; m.p. 121–123 °C; ^1^ H NMR (CDCl_3_, 500 MHz): *δ*_H_ 0.86 (s, Me-24), 0.87 (s, Me-23), 0.90 (s, Me-25), 0.94 (s, Me-26), 0.96 (s, Me-29), 1.17 (s, Me-27), 1.30 (s, Me-30), 1.91 (1 H, dd, *J* = 13.5 and 4.3 Hz, H-18), 3.15 (1 H, d, *J* = 10.9 Hz, H-28a), 3.48 (1 H, d, *J* = 10.9 Hz, H-28b), 4.44 (1 H, m, H-3), 5.12 (1 H, t, *J* = 3.6 Hz, H-12), 0.81 (t, *J* = 6.8 Hz, Me-16′), 1.54 (2 H, m, H-3′), 2.22 (2 H, t, *J* = 7.8 Hz, H-2′); ^13^ C NMR (CDCl_3_, 125 MHz): *δ*_C_ 38.3 (C-1), 23.6 (C-2), 80.5 (C-3), 37.8 (C-4), 55.2 (C-5), 18.2 (C-6), 32.5 (C-7), 39.8 (C-8), 47.6 (C-9), 36.8 (C-10), 23.5 (C-11), 122.3 (C-12), 144.2 (C-13), 41.7 (C-14), 25.5 (C-15), 22.0 (C-16), 36.9 (C-17), 42.3 (C-18), 46.4 (C-19), 30.9 (C-20), 34.1 (C-21), 31.0 (C-22), 28.0 (C-23), 16.7 (C-24), 15.6 (C-25), 16.8 (C-26), 25.9 (C-27), 69.7 (C-28), 33.2 (C-29), 23.6 (C-30), 173.7 (C-1′), 34.9 (C-2′), 25.2 (C-3′), 29.2–29.7 (C-4′-C-13′), 31.9 (C-14′), 22.7 (C-15′), 14.1 (C-16′).

**Erythrodiol (4)**: White powder; m.p. 231 °C; ^1^ H NMR (CDCl_3_, 500 MHz): *δ*_H_ 0.78 (s, Me-24), 0.80 (s, Me-23), 0.86 (s, Me-25), 0.87 (s, Me-26), 0.94 (s, Me-29), 0.99 (s, Me-27), 1.21 (s, Me-30), 1.91 (1 H, dd, *J* = 13.5 and 4.3 Hz, H-18), 3.15 (1 H, d, *J* = 10.8 Hz, H-28a), 3.48 (1 H, d, *J* = 10.8 Hz, H-28b), 3.48 (1 H, 1 H, dd, *J* = 11.5 and 4.6 Hz, H-3), 5.22 (1 H, t, *J* = 3.6 Hz, H-12); ^13^ C NMR (CDCl_3_, 125 MHz): *δ*_C_, 38.6 (C-1), 27.2 (C-2), 79.0 (C-3), 38.7 (C-4), 55.2 (C-5), 18.3 (C-6), 32.6 (C-7), 39.8 (C-8), 47.6 (C-9), 36.9 (C-10), 23.5 (C-11), 122.4 (C-12), 144.2 (C-13), 41.7 (C-14), 25.5 (C-15), 22.0 (C-16), 36.9 (C-17), 42.3 (C-18), 46.5 (C-19), 31.0 (C-20), 34.0 (C-21), 31.0 (C-22), 28.0 (C-23), 15.5 (C-24), 15.6 (C-25), 16.7 (C-26), 25.9 (C-27), 69.7 (C-28), 33.2 (C-29), 23.6 (C-30).

**3-*O*-acetylbetulin (5)**: White powder; m.p. 260-260.5 °C; ^1^ H NMR (CDCl_3_, 500 MHz): *δ*_H_ 0.72 (1 H, d, *J* = 10.6 Hz, H-5), 0.77 (s, Me-25), 0.79 (s, Me-23), 0.79 (s, Me-24), 0.90 (s, Me-27), 0.95 (s, Me-26), 1.22 (1 H, d, *J* = 2.7 Hz, H-9), 1.62 (s, Me-30), 2.32 (1 H, td, *J* = 10.9 and 5.8 Hz, H-19), 3.26 (1 H, d, *J* = 10.7 Hz, H-28a), 3.73 (1 H, d, *J* = 10.7 Hz, H-28b), 4.40 (1 H, dd, *J* = 10.9 and 5.4 Hz, H-3), 4.51 (1 H, d, *J* = 1.9 Hz, H-29a), 4.61 (1 H, d, *J* = 1.9 Hz, H-29b), 1.97 (s, Me-2′); ^13^ C NMR (CDCl_3_, 125 MHz): *δ*_C_ 38.4 (C-1), 23.7 (C-2), 80.9 (C-3), 37.8 (C-4), 55.4 (C-5), 18.2 (C-6), 34.2 (C-7), 40.9 (C-8), 50.3 (C-9), 37.0 (C-10), 20.8 (C-11), 25.2 (C-12), 37.3 (C-13), 42.7 (C-14), 27.0 (C-15), 29.2 (C-16), 47.8 (C-17), 48.7 (C-18), 47.8 (C-19), 150.5 (C-20), 29.7 (C-21), 33.9 (C-22), 27.9 (C-23), 15.9 (C-24), 16.5 (C-25), 16.2 (C-26), 14.7 (C-27), 60.6 (C-28), 109.7 (C-29), 19.1 (C-30), 171.0 (C-1′), 21.3 (C-2′).

**Betulinic acid (6)**: White powder; m.p. 277–279 °C; ^1^ H NMR (CDCl_3_, 500 MHz): *δ*_H_ 0.71 (1 H, d, *J* = 9.5 Hz, H-5), 0.78 (s, Me-24), 0.96 (s, Me-25), 0.99 (s, Me-23), 0.99 (s, Me-26), 1.00 (s, Me-27), 1.71 (s, Me-30), 2.34 (1 H, dd, *J* = 11.6 and 2.6 Hz, H-13), 3.02 (1 H, td, *J* = 10.8 and 4.9 Hz, H-18), 3.21 (1 H, dd, *J* = 11.4 and 4.8 Hz, H-3), 3.04 (1 H, dd, *J* = 11.1 and 4.8 Hz, H-19), 4.63 (s, H-29a), 4.76 (s, H-29b); ^13^ C NMR (CDCl_3_, 125 MHz): *δ*_C_ 38.7 (C-1), 27.4 (C-2), 79.0 (C-3), 38.9 (C-4), 55.3 (C-5), 18.3 (C-6), 34.3 (C-7), 40.7 (C-8), 50.5 (C-9), 37.2 (C-10), 20.9 (C-11), 25.5 (C-12), 38.4 (C-13), 42.4 (C-14), 30.6 (C-15), 32.2 (C-16), 56.3 (C-17), 46.9 (C-18), 49.3 (C-19), 150.4 (C-20), 29.7 (C-21), 37.0 (C-22), 28.0 (C-23), 15.3 (C-24), 16.0 (C-25), 16.1 (C-26), 14.7 (C-27), 180.4 (C-28), 109.7 (C-29), 19.4 (C-30).

**3-*O*-acetyllupeol (7)**: White powder; m.p. 216–218 °C; ^1^ H NMR (CDCl_3_, 500 MHz): *δ*_H_ 0.69 (1 H, d, *J* = 9.5 Hz, H-5), 0.77 (s, Me-28), 0.81 (s, Me-24), 0.82 (s, Me-23), 0.83 (s, Me-25), 0.92 (s, Me-27), 1.16 (s, Me-26), 1.66 (s, Me-30), 1.67 (dd, *J* = 11.6 and 2.6 Hz, H-13), 1.35 (1 H, td, *J* = 10.8 and 4.9 Hz, H-18), 2.35 (dd, *J* = 11.1 and 4.8 Hz, H-19), 4.40 (1 H, dd, *J* = 11.4 and 4.8 Hz, H-3), 4.55 (s, H-29a), 4.86 (s, H-29b), 2.03 (s, Me-2′); ^13^ C NMR (CDCl_3_, 125 MHz): *δ*_C_ 38.4 (C-1), 23.7 (C-2), 81.0 (C-3), 37.8 (C-4), 55.4 (C-5), 18.2 (C-6), 34.3 (C-7), 40.9 (C-8), 50.4 (C-9), 37.1 (C-10), 21.0 (C-11), 25.1 (C-12), 38.1 (C-13), 42.9 (C-14), 27.5 (C-15), 35.6 (C-16), 43.0 (C-17), 48.3 (C-18), 48.0 (C-19), 150.9 (C-20), 29.9 (C-21), 40.0 (C-22), 28.0 (C-23), 16.5 (C-24), 16.2 (C-25), 16.0 (C-26), 14.5 (C-27), 18.0 (C-28), 109.3 (C-29), 19.3 (C-30), 170.8 (C-1′), 21.3 (C-2′).

**Lupeol (8)**: White powder; m.p. 212–214 °C; ^1^ H NMR (CDCl_3_, 500 MHz): *δ*_H_ 0.78 (s, Me-25), 0.79 (s, Me-24), 0.86 (s, Me-26), 0.95 (s, Me-27), 0.98 (s, Me-23), 1.71 (s, Me-30), 2.40 (1 H, td, *J* = 11.1 and 5.8 Hz, H-19), 3.21 (1 H, dd, *J* = 11.4 and 4.9 Hz, H-3), 4.59 (1 H, d, *J* = 2.3 Hz, H-29a), 4.71 (1 H, d, *J* = 2.3 Hz, H-29b); ^13^ C NMR (CDCl_3_, 125 MHz): *δ*_C_ 38.6 (C-1), 27.6 (C-2), 79.0 (C-3), 38.6 (C-4), 55.3 (C-5), 18.2 (C-6), 33.2 (C-7), 41.9 (C-8), 50.2 (C-9), 37.4 (C-10), 20.6 (C-11), 23.7 (C-12), 32.5 (C-13), 42.5 (C-14), 27.5 (C-15), 40.4 (C-16), 48.6 (C-17), 53.8 (C-18), 48.0 (C-19), 151.0 (C-20), 27.6 (C-21), 44.5 C-22), 28.2 (C-23), 15.7 (C-24), 16.8 (C-25), 16.1 (C-26), 15.3 (C-27), 16.8 (C-28), 109.3 (C-29), 19.4 (C-30).

**Myrianthic acid (9)**: White powder, m.p. > 300 °C; ^1^ H NMR (acetone-*d*_*6*_, 500 MHz): *δ*_H_ 0.75 (s, Me-26), 0.78 (s, Me-24), 0.91 (d, *J* = 6.7 Hz, Me-30), 0.99 (s, Me-25), 1.18 (s, Me-29), 1.34 (s, Me-27), 1.49 (1 H, m, H-16a), 2.50 (1 H, s, H-18), 2.60 (1 H, td, *J* = 13.3 and 4.6 Hz, H-16b), 3.37 (1 H, d, *J* = 11.0 Hz, 23a), 3.50 (1 H, d, *J* = 11.0 Hz, 23b), 3.60 (1 H, d, *J* = 2.5 Hz, H-3), 3.89 (1 H, m, H-2), 5.27 (1 H, t, *J* = 3.5 Hz, H-12); ^13^ C NMR (acetone-*d*_*6*_, 125 MHz): *δ*_C_ 41.0 (C-1), 65.6 (C-2), 77.7 (C-3), 41.4 (C-4), 42.6 (C-5), 17.7 (C-6), 32.4 (C-7), 41.1 (C-8), 47.0 (C-9), 37.7 (C-10), 23.4 (C-11), 127.7 (C-12), 138.8 (C-13), 39.8 (C-14), 28.3 (C-15), 25.3 (C-16), 47.3 (C-17), 53.5 (C-18), 72.1 (C-19), 41.8 (C20), 26.0 (C-21), 37.6 (C-22), 70.0 (C-23), 16.3 (C-24), 16.1 (C-25), 16.5 (C-26), 23.7 (C-27), 179.5 (C-28), 26.0 (C-29), 15.5 (C-30).

**Pomolic acid (10)**: White powder; m.p. 272–274 °C; ^1^ H NMR (CD_3_OD, 500 MHz): *δ*_H_ 0.92 (s, Me-23), 1.03 (s, Me-24), 1.07 (s, Me-25), 1.08 (s, Me-26), 1.14 (d, *J* = 6.9 Hz, Me-30), 1.28 (s, Me-27), 1.90 (s, Me-29), 2.62 (1 H, s, H-18), 3.47 (1 H, dd, *J* = 11.2 and 4.1 Hz, H-3), 5.75 (1 H, t, *J* = 3.6 Hz, H-12); ^13^ C NMR (CD_3_OD, 125 MHz): *δ*_C_ 38.7 (C-1), 27.5 (C-2), 79.1 (C-3), 38.5 (C-4), 55.3 (C-5), 18.4 (C-6), 32.8 (C-7), 40.0 (C-8), 47.3 (C-9), 36.9 (C-10), 23.7 (C-11), 129.1 (C-12), 138.0 (C-13), 41.1 (C-14), 28.2 (C-15), 25.5 (C-16), 47.9 (C-17), 53.2 (C-18), 73.1 (C-19), 41.1 (C-20), 26.0 (C-21), 37.5 (C-22), 28.2 (C-23), 15.2 (C-24), 15.5 (C-25), 16.6 (C-26), 24.6 (C-27), 180.6 (C-28), 27.3 (C-29), 16.2 (C-30).

**Ursolic acid (11)**: White powder; m.p. 284 °C; ^1^ H NMR (C_5_D_5_N, 500 MHz): *δ*_H_ 0.86 (1 H, d, *J* = 11.9 Hz, H-5), 0.95 (d, *J* = 6.2 Hz, Me-30), 1.00 (d, *J* = 6.4 Hz, Me-29), 1.22 (s, Me-27), 1.24 (s, Me-23), 1.55 (2 H, dd, *J* = 14.0 and 6.5 Hz, H-1), 1.63 (1 H, dd, *J* = 10.1 and 7.6 Hz, H-9), 2.12 (2 H, td, *J* = 13.3 and 4.2 Hz, H-16), 2.33 (2 H, td, *J* = 13.4 and 4.6 Hz, H-15), 2.63 (1 H, d, *J* = 11.3 Hz, H-18), 3.45 (1 H, dd, *J* = 10.1 and 5.8 Hz, H-3), 5.49 (1 H, t, *J* = 3.3 Hz, H-12); ^13^ C NMR (C_5_D_5_N, 125 MHz): *δ*_C_ 38.9 (C-1), 27.9 (C-2), 77.9 (C-3), 39.2 (C-4), 55.6 (C-5), 18.6 (C-6), 33.4 (C-7), 39.7 (C-8), 47.8 (C-9), 37.1 (C-10), 23.4 (C-11), 125.4 (C-12), 139.1 (C-13), 42.3 (C-14), 28.5 (C-15), 24.7 (C-16), 47.8 (C-17), 53.3 (C-18), 39.3 (C-19), 39.2 (C-20), 30.9 (C-21), 37.2 (C-22), 28.6 (C-23), 17.3 (C-24), 15.5 (C-25), 17.2 (C-26), 23.7 (C-27), 179.7 (C-28), 16.4 (C-29), 21.2 (C-30).

**Taraxerone (12)**: White powder; m.p. 238–240 °C; ^1^ H NMR (CDCl_3_, 500 MHz): *δ*_H_ 0.76 (s, Me-29), 0.84 (s, Me-30), 0.85 (s, Me-23), 0.89 (s, Me-24), 1.00 (s, Me-25), 1.01 (s, Me-26), 1.02 (s, Me-28), and 1.07 (s, Me-27), 1.85 (1 H, dd, *J* = 14.8 and 3.1 Hz, H-16a), 2.01 (1 H, dt, *J* = 12.9 and 3.3 Hz, H-16b), 2.26 (1 H, ddd; *J* = 15.8; 6.4 and 3.3 Hz, H-2a), 2.51 (1 H, ddd; *J* = 15.8; 11.8 and 7.1 Hz, H-2b), 5.49 (1 H, dd, *J* = 8.2 and 3.2 Hz, H-15); ^13^ C NMR (CDCl_3_, 125 MHz): *δ*_C_ 38.3 (C-1), 34.1 (C-2), 217.5 (C-3), 47.6 (C-4), 55.8 (C-5), 19.9 (C-6), 35.1 (C-7), 38.9 (C-8), 48.7 (C-9), 35.8 (C-10), 17.4 (C-11), 37.7 (C-12), 37.7 (C-13), 157.6 (C-14), 117.2 (C-15), 36.6 (C-16), 37.5 (C-17), 48.8 (C-18), 40.6 (C-19), 28.8 (C-20), 33.6 (C-21), 33.1 (C-22), 26.1 (C-23), 21.5 (C-24), 14.8 (C-25), 29.9 (C-26), 25.6 (C-27), 29.8 (C-28), 33.3 (C-29), 21.3 (C-30).

**22,23-Dihydrospinasterol 3-*O*-*β*-*D*-glucopyranoside (13)**: White powder; m.p. 284 °C; ^1^ H NMR (DMSO-*d*_*6*_, 500 MHz): *δ*_H_ 0.48 (s, Me-18), 0.72 (s, Me-19), 0.89 (d, *J* = 5.9 Hz, Me-21), 0.90 (1 H, m, H-24), 3.53 (1 H br s, H-3), 5.10 (1 H br s, H-7), 3.00 (1 H br s, H-4′), 3.09 (1 H br s, H-3′), 3.09 (1 H br s, H-5′), 3.40 (1 H, m, H-6′a), 3.62 (1 H, br d, *J* = 5.9 Hz, H-6′b), 3.87 (1 H br s, H-2′), 4.20 (1 H, d, *J* = 7.1 Hz, H-1′); ^13^ C NMR (DMSO-*d*_*6*_, 500 MHz): *δ*_C_ 37.0 (C-1), 31.8 (C-2), 76.7 (C-3), 43.4 (C-4), 40.5 (C-5), 29.6 (C-6), 117.6 (C-7), 139.5 (C-8), 49.1 (C-9), 34.4 (C-10), 21.4 (C-11), 39.3 (C-12), 43.3 (C-13), 54.8 (C-14), 23.0 (C-15), 28.3 (C-16), 55.9 (C-17), 12.1 (C-18), 13.2 (C-19), 36.4 (C-20), 19.3 (C-21), 33.8 (C-22), 26.0 (C-23), 45.6 (C-24), 29.2 (C-25), 19.4 (C-26), 19.2 (C-27), 23.1 (C-28), 12.3 (C-29). 101.1 (C-1′), 73.9 (C-2′), 77.1 (C-3′), 70.6 (C-4′), 77.2 (C-5′), 61.6 (C-6′).

**Spinasterol (14)**: White powder; m.p. 171–173 °C; ^1^ H NMR (CDCl_3_, 500 MHz): *δ*_H_ 0.48 (s, Me-18), 0.73 (s, Me-19), 0.96 (d, *J* = 6.6 Hz, Me-21), 1.32 (1 H, dd, *J* = 11.3 and 4.0 Hz, H-5), 1.46 (1 H, m, H-24), 1.90 (1 H, d, *J* = 2.9 Hz, H-12a), 1.94 (1 H, d, *J* = 4.3 Hz, H-12b), 1.95 (1 H, m, H-20), 3.53 (1 H t, *J* = 4.5 Hz, H-3), 4.96 (1 H dd, *J* = 15.1 and 8.6 Hz, H-23), 5.09 (1 H, m, H-7), 5.09 (1 H, m, H-22); ^13^ C NMR (CDCl_3_, 500 MHz): *δ*_C_ 36.8 (C-1), 31.2 (C-2), 71.0 (C-3), 37.9 (C-4), 40.1 (C-5), 29.6 (C-6), 117.4 (C-7), 138.2 (C-8), 49.5 (C-9), 34.0 (C-10), 21.2 (C-11), 39.4 (C-12), 43.2 (C-13), 55.4 (C-14), 22.9 (C-15), 28.5 (C-16), 55.7 (C-17), 12.0 (C-18), 13.0 (C-19), 40.7 (C-20), 21.2 (C-21), 138.1 (C-22), 129.4 (C-23), 51.2 (C-24), 31.8 (C-25), 19.0 (C-26), 21.2 (C-27), 25.3 (C-28), 12.0 (C-29).

**Spinasterol 3-*O*-*β*-*D*-glucopyranoside (15)**: White powder; m.p. 279–281 °C; ^1^ H NMR (C_5_D_5_N, 500 MHz): *δ*_H_ 0.51 (s, Me-18), 0.73 (s, Me-19), 0.77 (m, Me-26), 0.77 (m, Me-29), 0.82 (d, *J* = 6:5 Hz; Me-27), 0.99 (d, *J* = 6:5 Hz; Me-21), 3.54 (1 H, m, H-3), 5.02 (1 H, dd, *J* = 9:0; 15.0 Hz, H-23), 5.11 (1 H, m, H-7), 5.16 (1 H, dd, *J* = 9:0; 15.0 Hz, H-22), 2.88 (1 H, t, *J* = 9:0 Hz, H-5′), 3.05 (1 H, overlapped, H-2′), 3.05 (1 H, overlapped, H-3′), 3.05 (1 H, overlapped, H-4′), 3.40 (1 H, dd, *J* = 5:0; 11.0 Hz, H-6′a), 3.63 (1 H, d, *J* = 11:0; H-6′b), 4.21 (1 H, d, *J* = 8:0 Hz; H-1′); ^13^ C NMR (C_5_D_5_N, 500 MHz): *δ*_C_ 37.3 (C-1), 30.0 (C-2), 77.1 (C-3), 34.6 (C-4), 40.2 (C-5), 30.0 (C-6), 117.9 (C-7), 139.6 (C-8), 49.6 (C-9), 34.8 (C-10), 21.8 (C-11), 39.6 (C-12), 43.5 (C-13), 55.3 (C-14), 23.4 (C-15), 29.0 (C-16), 56.1 (C-17), 12.3 (C-18), 13.1 (C-19), 41.2 (C-20), 21.7 (C-21), 138.7 (C-22), 129.7 (C-23), 51.5 (C-24), 32.2 (C-25), 19.2 (C-26), 21.3 (C-27), 25.7 (C-28), 12.6 (C-29), 102.3 (C-1′), 75.4 (C-2′), 78.7 (C-3′), 71.8 (C-4′), 78.6 (C-5′), 62.9 (C-6′).

**Stigmasterol (16)**: White powder; m.p. 175–177 °C; ^1^ H NMR (CDCl_3_, 500 MHz): *δ*_H_ 0.74 (s, Me-19), 0.81 (d, *J* = 6.7 Hz, Me-26), 0.83 (d, *J* = 6.7 Hz, Me-27), 0.86 (t, *J* = 7.2 Hz, Me-28), 0.92 (d, *J* = 6.5 Hz, Me-21), 1.05 (s, Me-18); 3.53 (1 H, tdd, *J* = 4.6, 4.5 and 3.7 Hz, H-3), 4.98 (1 H, m, H-22), 5.14 (1 H, m, H-23), 5.34 (1 H, t, *J* = 6.5 Hz, H-5); ^13^ C NMR (CDCl_3_, 125 MHz): *δ*_C_ 37.2 (C-1), 31.9 (C-2), 71.8 (C-3), 42.3 (C-4), 140.8 (C-5), 121.7 (C-6), 31.7 (C-7), 31.9 (C-8), 50.2 (C-9), 36.5 (C-10), 21.1 (C-11), 39.7 (C-12), 42.2 (C-13), 56.9 (C-14), 24.4 (C-15), 28.9 (C-16), 56.0 (C-17), 19.4 (C-18), 12.1 (C-19), 40.5 (C-20), 21.1 (C-21), 138.3 (C-22), 129.3 (C-23), 51.3 (C-24), 31.9 (C-25), 21.2 (C-26), 19.0 (C-27), 25.4 (C-28), 12.3 (C-29).

**Lacourtianamide (17)**: White powder; ^1^ H NMR (C_5_D_5_N, 500 MHz): *δ*_H_ 0.85 (t, *J* = 5.6 Hz, Me-25), 1.13–1.48 (br s, H-11-H-22), 1.26 (2 H, m, H-24), 1.27 (2 H, m, H-23), 1.27 (2 H, m, H-6), 1.92 (1 H, m, H-5a), 1.99 (2 H, m, H-10), 2.17 (2 H, m, H-7), 2.24 (1 H, m, H-5b), 4.29 (1 H, m, H-4), 4.36 (1 H, m, H-3), 4.43 1 H, dd, *J* = 9.8 and 5.0 Hz, H-1a), 4.52 (1 H, m, H-1b), 5.12 (1 H, m, H-2), 5.49 (1 H, dt, *J* = 15.4 and 6.4 Hz, H-9), 5.54 (1 H, dt, *J* = 15.4 and 6.4 Hz, H-8), 8.58 (1 H, d, *J* = 9.0 Hz, N-H), 0.85 (t, *J* = 5.6 Hz, Me-19′), 1.13–1.48 (br s, H-5′-H-16′), 1.26 (2 H, m, H-18′), 1.27 (2 H, m, H-17′), 1.75 (2 H, m, H-4′), 2.02 (1 H, m, H-3′a), 2.25 (1 H, m, H-3′b), 4.62 (1 H, br s, H-2′); ^13^ C NMR (C_5_D_5_N, 125 MHz): *δ*_C_ 61.8 (C-1), 52.7 (C-2), 76.6 (C-3), 72.8 (C-4), 33.9 (C-5), 26.4 (C-6), 33.1 (C-7), 130.7 (C-8), 130.6 (C-9), 32.8 (C-10), 29.3–30.1 (C11-C-22), 31.9 (C-23), 22.7 (C-24), 14.1 (C-25), 175.0 (C-1′), 72.2 (C-2′), 35.5 (C-3′), 25.6 (C-4′), 29.3–30.1 (C-5′-C-16′), 31.9 (C-17′), 22.7 (C-18′), 14.1 (C-19′).

**Lacourtianoside II (18)** Yellow powder; ^1^ H NMR (CDCl_3_/CD_3_OD 1:1, 500 MHz): *δ*_H_ 0.87 (3 H, t, *J* = 6.9 Hz, H-37), 4.06 (1 H, m, H-1a), 3.21 (1 H, m, H-3′ ), 3.30 (1 H, br d, *J* = 6.9 Hz, H-4′) 3.39 (1 H, m, H-5′ ), 3.70 (1 H, dd, *J* = 11.9 and 4.8 Hz, H-6′b), 3.82 (1 H, m, H-1b), 3.87 (1 H, dd, *J* = 11.9 and 1.9 Hz, H-6′a), 4.03 (1 H, dd, *J =* 7.8 and 3.6 Hz, H-2′ ), 4.27 (1 H, d, *J* = 7.8 Hz, H-1′); ^13^ C NMR (CDCl_3_/CD_3_OD 1:1, 125 MHz): *δ*_C_ 68.5 (C-1), 34.3 (C-2), 29.5–29.2 (C-3-C-35), 31.8 (C-35), 22.5 (C-36), 37 (C-37), 103.1 (C-1′), 71.8 (C-2′), 73.4 (C-3′), 70.0 (C-4′), 76.4 (C-5′), 61.4 (C-6′).

**Epicatechin (19)**: White amorphous powder; m.p. 235–237 °C; ^1^ H NMR (CD_3_OD, 500 MHz): *δ*_H_ 2.77 (1 H, dd, *J* = 16.7 and 2.5 Hz, H-4b), 2.86 (1 H, dd, *J* = 16.7 and 4.6 Hz, H-4a), 4.17 (1 H, br s, H-3), 4.81 (1 H, br s, H-2), 5.91 (1 H, d, *J* = 2.1 Hz, H-8), 5.94 (1 H, d, *J* = 2.1 Hz, H-6), 6.75 (1 H, d, *J* = 8.1 Hz, H-5′), 6.79 (1 H, dd, *J* = 8.1 and 2.0 Hz, H-6′), 6.97 (1 H, d, *J* = 2.0 Hz, H-2′); ^13^ C NMR (CD_3_OD, 125 MHz): *δ*_C_ 78.5 (C-2), 66.1 (C-3), 27.8 (C-4), 98.7 (C-4a), 156.3 (C-5), 95.0 (C-6), 156.6 (C-7), 94.5 (C-8), 155.9 (C-8a), 130.9 (C-1′), 113.9 (C-2′), 144.5 (C-3′), 144.4 (C-4′), 114.5 (C-5′), 118.0 (C-6′).

**Methyl pheophorbide-a (20)**: Blue amorphous powder; m.p. 226–228 °C; ^1^ H NMR (CDCl_3_, 500 MHz): *δ*_H_ 1.70 (t; *J* = 7.6 Hz, Me-8^2^), 3.20 (s, Me-7^1^), 3.41 (s, Me-2^1^), 3.61 (s, MeO-17^5^), 3.65 (2 H, q, *J* = 7.6, H-8^1^), 3.70 (s, Me-12^1^), 3.92 (s, MeO-13^5^), 4.24 (1 H, dt, *J* = 9.0 and 2.6 Hz, H-17), 6.18 (1 H, dd, *J* = 11.5 and 1.5 Hz, H-3^2^), 6.27 (1 H, dd, *J* = 17.9 and 1.5 Hz, H-3^2^′), 7.97 (1 H, dd, *J* = 17.9 and 11.5 Hz, H-3^1^), 8.58 (1 H, s, H-20), 9.34 (1 H, s, H-5), 9.49 (1 H, s, H-10); ^13^ C NMR (CDCl_3_, 125 MHz): *δ*_C_ 142.1 (C-1), 131.9 (C-2), 12.1 (C-2^1^), 136.2 (C-3), 128.9 (C-3^1^), 122.8 (C-3^2^), 136.3 (C-4), 97.6 (C-5), 155.7 (C-6), 136.5 (C-7), 11.2 (C-7^1^), 145.2 (C-8), 19.5 (C-8^1^), 17.4 (C-8^2^), 151.0 (C-9), 104.5 (C-10), 137.9 (C-11), 129.1 (C-12), 12.1 (C-12^1^), 128.9 (C-13), 189.6 (C-13^1^), 64.7 (C-13^2^), 169.6 (C-13^3^), 52.9 (C-13^5^), 149.7 (C-14), 105.2 (C-15), 161.2 (C-16), 51.1 (C-17), 29.4 (C-17^1^), 23.1 (C-17^2^), 173.3 (C-17^3^), 51.7 (C-17^5^), 50.1 (C-18), 22.7 (C-18^1^),172.2 (C-19), 93.1 (C-20).

## Results

Violacein production, swarming motility and biofilm formation are amongst the important quorum-sensing mediated processes in bacteria. The disruption of quorum-sensing communication networks in bacterial colonies is an effective strategy to eliminate or reduce resistance to antibiotics and it is not intended to kill bacteria but to prevent the expression of their virulence factors and pathogenicity [27]. For this reason, violacein inhibition, anti-QS, swarming inhibition and antibiofilm assays were performed at concentrations below the minimal inhibitory concentration (sub-MIC).

### Inhibition of violacein production in C. violaceum CV12472

When *Chromobacterium violaceum* grows, it produces a violet pigment called violacein which plays the role of a signal molecule and indicates proper and normal functioning of this bacterium. Violacein production is mediated by QS process in *C. violaceum* CV12472. The inhibition of violacein at sub-MIC concentrations is significant as it indicates the potential of the compounds to prevent signal molecule production in bacteria. Prior to evaluation of violacein inhibition, MIC values were determined and almost all compounds inhibited *C. violaceum* CV12472 within test concentrations except Lacourtianoside II as shown on Table 1. For the active compounds, MIC values varied from 0.25 mg/mL for the most active compound Epicatechin to 1.00 mg/mL. Epicatechin had the highest violacein inhibition of 100% at MIC and was the only compound to inhibit violacein production at MIC/8 with percentage inhibition of 17.2 ± 0.9%. Some other compounds were also active at MIC/4 concentration including Acetylerythrodiol (14.2 ± 0.28%), Pomolic acid (9.7 ± 0.2%), Ursolic acid (7.5 ± 0.4%), Lacourtianamide (5.5 ± 0.1%), Epicatechin (39.8 ± 0.6%) and Methyl pheophorbide-a (8.4 ± 0.5%). All compounds inhibited violacein at MIC except compound 18 and at MIC/2 except compounds 22,23-Dihydrospinasterol 3-O-β-D-glucopyranoside, Spinasterol 3-O-β-D-glucopyranoside and Lacourtianoside II.

**Table 1:** Inhibition of violacein production in *C. violaceum* CV12472 by compounds

Compound	MIC (mg/mL)	Violacein inhibition (%)			
		**MIC**	**MIC/2**	**MIC/4**	**MIC/8**
Acetylerythrodiol	1.00	45.5 ± 1.5	26.0 ± 0.6	14.2 ± 0.28	-
Amyrincapraote	0.50	29.3 ± 0.2	10.5 ± 0.1	-	-
Erythrodiol 3-O-palmitate	0.50	18.5 ± 0.5	07.9 ± 0.3	-	-
Erythrodiol	1.00	37.3 ± 0.8	13.6 ± 0.1	-	-
3-O-acetylbetulin	1.00	60.1 ± 1.0	21.9 ± 0.6	-	-
Betulinic acid	0.50	26.9 ± 0.1	15.1 ± 0.4	-	-
3-O-acetyllupeol	1.00	27.5 ± 1.1	10.0 ± 0.5	-	-
Lupeol	1.00	38.0 ± 0.4	12.9 ± 0.2	-	-
Myrianthic acid	1.00	25.8 ± 0.7	10.3 ± 0.4	-	-
Pomolic acid	0.50	44.2 ± 0.22	28.0 ± 0.5	9.7 ± 0.2	-
Ursolic acid	1.00	60.1 ± 1.0	21.9 ± 0.6	7.5 ± 0.4	-
Taraxerone	0.50	25.9 ± 0.6	11.0 ± 0.3	-	-
22,23-Dihydrospinasterol 3-O-β-D-glucopyranoside	1.00	20.5 ± 0.3	-	-	-
Spinasterol	1.00	20.9 ± 0.10	11.3 ± 0.5	-	-
Spinasterol 3-O-β-D-glucopyranoside	1.00	17.49 ± 0.6	-	-	-
Stigmasterol	1.00	22.9 ± 0.8	06.7 ± 0.2	-	-
Lacourtianamide	0.50	43.8 ± 1.5	19.7 ± 0.4	5.5 ± 0.1	-
Lacourtianoside II	> 1.00	-	-	-	-
Epicatechin	0.25	100 ± 0.00	86.7 ± 1.7	39.8 ± 0.6	17.2 ± 0.9
Methyl pheophorbide-a	0.50	41.0 ± 1.0	22.6 ± 0.5	8.4 ± 0.5	-

*-: No inhibition; values are means ± SEM for three parallel measurements*

### Quorum sensing inhibition in mutant strain C. violaceum CV026

**Fig. 3:**
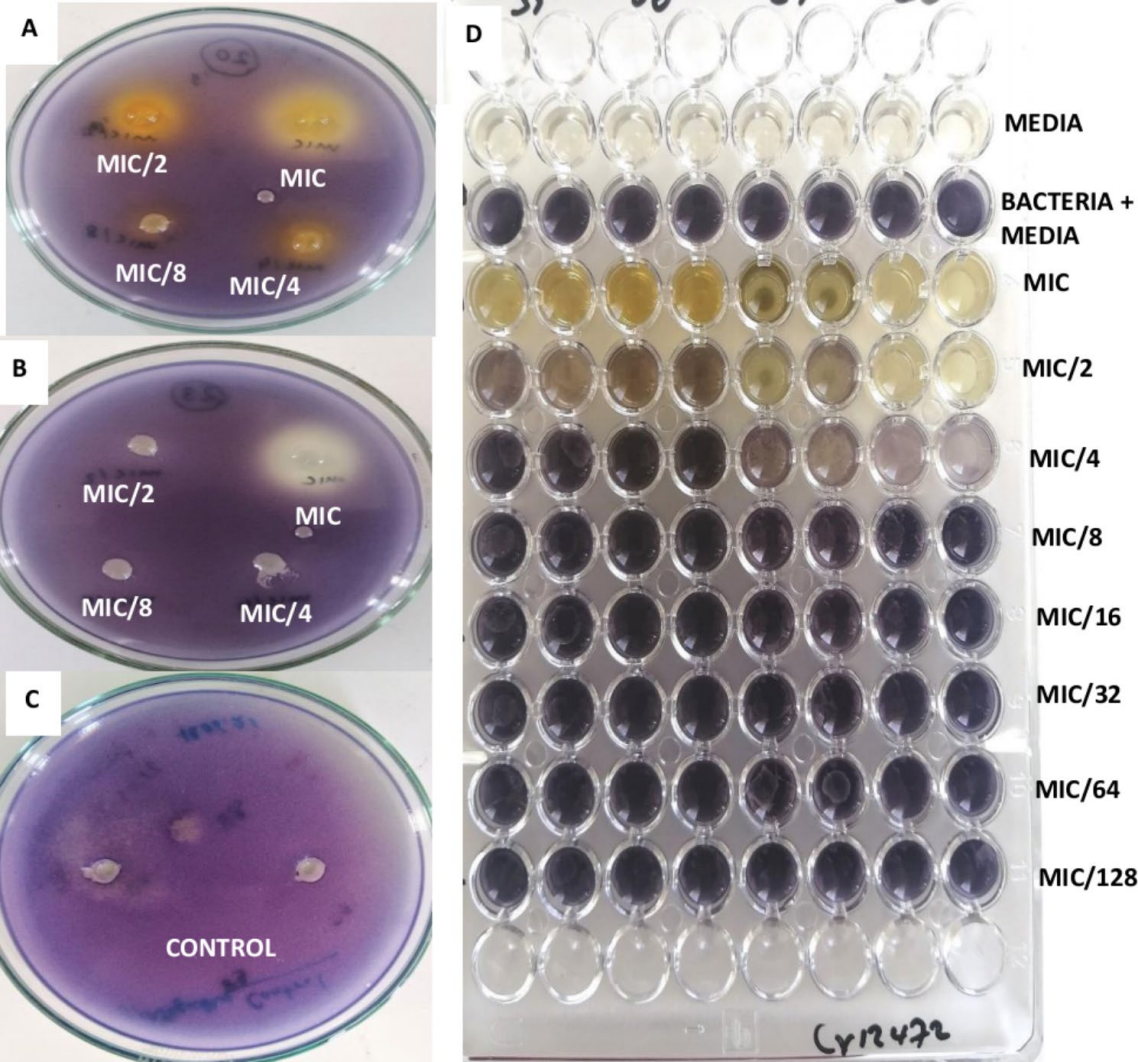
Quorum-sensing inhibition plates (**A** and **B**); Quorum-sensing control plate (**C**); violacein inhibition plate (**D**)

The mutant strain *C. violaceum* 026 does not produce violacein when growing unless if an acylhomoserine lactone (AHL) is supplied to it from an external source. However, in this assay the hormone is supplied in the presence of the test compounds so as to evaluate the ability of the compounds to prevent the response of the bacteria cells to the AHL. This assay was done at sub-MIC concentrations and the zones of inhibition in millimetres are reported on Table 2. Erythrodiol, Lupeol, Taraxerone, 22,23-Dihydrospinasterol 3-O-β-D-glucopyranoside, Spinasterol, Spinasterol 3-O-β-D-glucopyranoside, Stigmasterol and Lacourtianoside II could not inhibit *C. violaceum* CV026 within the tested concentrations while the other compounds inhibited this bacterium with MIC values varying from 0.50 mg/mL to 1.00 mg/mL. No compound showed anti-QS activity at MIC/8 and only the most active compound, Epicatechin showed inhibition of QS with inhibition zones of 9.5 ± 0.1 mm at MIC/4. At MIC/2, Acetylerythrodiol (9.1 ± 0.4 mm), Betulinic acid (8.5 ± 0.2 mm), Myrianthic acid (9.8 ± 0.4 mm), Pomolic acid (10.5 ± 0.7 mm), Ursolic acid (11.3 ± 0.5 mm), Lacourtianamide (9.1 ± 0.5 mm), Epicatechin (11.5 ± 0.5 mm) and Methyl pheophorbide-a (10.3 ± 0.1 mm) were able to inhibit QS as per the inhibition zones shown on Fig. 3. However, at MIC concentration, most of the compounds inhibited QS.

**Table 2:** Quorum sensing inhibition zones in *C. violaceum* CV026 by compounds

Compound	Anti-quorum sensing inhibition zones (mm)			
	**MIC (mg/mL)**	**MIC**	**MIC/2**	**MIC/4**
Acetylerythrodiol	0.50	12.0 ± 1.0	9.1 ± 0.4	-
Amyrincapraote	1.00	9.1 ± 0.5	-	-
Erythrodiol 3-O-palmitate	1.00	7.5 ± 0.2	-	-
Erythrodiol	> 1.00	-	-	-
3-O-acetylbetulin	0.50	11.5 ± 0.5	-	-
Betulinic acid	1.00	11.0 ± 0.2	8.5 ± 0.2	-
3-O-acetyllupeol	1.00	9.0 ± 0.5	-	-
Lupeol	1.00	-	-	-
Myrianthic acid	0.50	12.0 ± 1.0	9.8 ± 0.4	-
Pomolic acid	0.50	13.0 ± 0.5	10.5 ± 0.7	-
Ursolic acid	1.00	14.0 ± 1.0	11.3 ± 0.5	-
Taraxerone	1.00	-	-	-
22,23-Dihydrospinasterol 3-O-β-D-glucopyranoside	> 1.00	-	-	-
Spinasterol	> 1.00	-	-	-
Spinasterol 3-O-β-D-glucopyranoside	> 1.00	-	-	-
Stigmasterol	> 1.00	-	-	-
Lacourtianamide	0.50	13.5 ± 0.5	9.1 ± 0.5	-
Lacourtianoside II	> 1.00	-	-	-
Epicatechin	0.50	16.0 ± 1.0	11.5 ± 0.5	9.5 ± 0.1
Methyl pheophorbide-a	1.00	13.0 ± 1.0	10.3 ± 0.1	-

*-: No inhibition; values are means ± SEM for three parallel measurements*

### Inhibition of swarming motility on P. aeruginosa PA01

*P. aeruginosa* PA01 is an opportunistic bacterium that inhabits many environments capable of utilizing several motility strategies to move and colonize surfaces and establishing biofilms. Swarming motility constitutes an important process in this bacterium and for this reason, inhibiting swarming is a good strategy to eliminate its pathogenicity. The inhibition of swarming movement in *P. aeruginosa* PA01 was carried out at 100, 75 and 50 µg/mL and reported on Table 3. All the compounds showed swarming inhibitions at 100 µg/mL concentration and this activity further varied in a concentration dependent manner at 75 and 50 µg/mL. The most active compound was Epicatechin whose anti-swarming activity varied from 58.84 ± 1.28% at 100 µg/mL to 20.52 ± 0.14% at 50 µg/mL. Pomolic acid also exhibited good activity at 100 µg/mL (51.61 ± 1.24%) and at 50 µg/mL (22.64 ± 0.44%) as well as Ursolic acid at 100 µg/mL (54.74 ± 1.08%) and at 50 µg/mL (15.92 ± 0.46%) and Lacourtianamide at 100 µg/mL (50.94 ± 1.05%) and at 50 µg/mL (17.52 ± 0.34%). Acetylerythrodiol, Amyrincapraote, Erythrodiol 3-O-palmitate, 3-O-acetylbetulin, 3-O-acetyllupeol, 22,23-Dihydrospinasterol 3-O-β-D-glucopyranoside, Spinasterol 3-O-β-D-glucopyranoside, Lacourtianoside II had weak activities as they only inhibited swarming at MIC.

**Table 3:** Swarming motility inhibition on *P. aeruginosa* PA01 by compounds

Compound	Swarming inhibition (%)		
	**100 µg/mL**	**75 µg/mL**	**50 µg/mL**
Acetylerythrodiol	10.59 ± 0.24	-	-
Amyrincapraote	19.85 ± 0.70	-	-
Erythrodiol 3-O-palmitate	12.90 ± 0.32	-	-
Erythrodiol	45.10 ± 0.11	28.52 ± 0.37	14.40 ± 0.05
3-O-acetylbetulin	11.19 ± 0.36	-	-
Betulinic acid	40.94 ± 1.05	21.22 ± 0.57	10.32 ± 0.21
3-O-acetyllupeol	11.50 ± 0.28	-	-
Lupeol	29.35 ± 0.80	12.72 ± 0.26	-
Myrianthic acid	33.81 ± 0.64	19.15 ± 0.25	13.11 ± 0.04
Pomolic acid	51.61 ± 1.24	35.85 ± 0.67	22.64 ± 0.44
Ursolic acid	54.74 ± 1.08	40.25 ± 0.65	15.92 ± 0.46
Taraxerone	24.24 ± 0.15	15.25 ± 0.32	11.38 ± 0.04
22,23-Dihydrospinasterol 3-O-β-D-glucopyranoside	13.75 ± 0.35	-	-
Spinasterol	25.15 ± 0.30	10.38 ± 0.16	-
Spinasterol 3-O-β-D-glucopyranoside	13.06 ± 0.10	-	-
Stigmasterol	21.38 ± 0.50	6.14 ± 0.05	-
Lacourtianamide	50.94 ± 1.05	31.42 ± 0.85	17.52 ± 0.34
Lacourtianoside II	15.26 ± 0.31	-	-
Epicatechin	58.84 ± 1.28	38.45 ± 0.68	20.52 ± 0.14
Methyl pheophorbide-a	22.52 ± 0.35	9.60 ± 0.2	-

*-: No inhibition; values are means ± SEM for three parallel measurements*

### Antimicrobial activity

The antimicrobial effects of the compounds were evaluated on three strains: one Gram-positive (*S. aureus*), one Gram-negative (*E. coli*) and one yeast (*C. albicans*) and the minimal inhibitory concentration of each compound on each of the microorganisms are given on Table 4. The Gram-positive bacteria *S. aureus* was susceptible to all the compounds except to 22,23-Dihydrospinasterol 3-O-β-D-glucopyranoside, Spinasterol 3-O-β-D-glucopyranoside, Stigmasterol and Lacourtianoside II. The other compounds had MIC values on *S. aureus* varying from 0.25 mg/mL for the most active compound, Lacourtianamide to 1.00 mg/mL for Acetylerythrodiol, 3-O-acetyllupeol, Pomolic acid, Ursolic acid, Spinasterol and Methyl pheophorbide-a. All other compounds had MIC values on *S. aureus* to be 0.50 mg/mL. The Gram-negative bacteria *E. coli* was the least susceptible to the compounds and only Epicatechin had a MIC value of 0.50 mg/mL against *E. coli*, while the other active compounds Acetylerythrodiol, Amyrincapraote, Erythrodiol 3-O-palmitate, 3-O-acetylbetulin, Betulinic acid, 3-O-acetyllupeol, Myrianthic acid, Ursolic acid, Lacourtianamide, Methyl pheophorbide-a, all had MIC values of 1.00 mg/mL. The other compounds could not inhibit *E. coli* within the tested concentrations. Against the yeast cells *C. albicans*, Erythrodiol 3-O-palmitate, Erythrodiol and 3-O-acetylbetulin were not active within tested concentrations. The most active compounds against *C. albicans* were Acetylerythrodiol, 3-O-acetyllupeol, Pomolic acid, Ursolic acid, Lacourtianamide, Epicatechin and Methyl pheophorbide-a which had MIC values of 0.50 mg/mL. The other compounds inhibited *C. albicans* with MIC values of 1.00 mg/mL.

**Table 4:** Antimicrobial activity (MIC values in mg/mL) of compounds

Compound	Microorganism		
	***S. aureus***	***E. coli***	***C. albicans***
Acetylerythrodiol	1.00	1.00	0.50
Amyrincapraote	0.50	1.00	1.00
Erythrodiol 3-O-palmitate	0.50	1.00	> 1.00
Erythrodiol	0.50	> 1.00	> 1.00
3-O-acetylbetulin	0.50	1.00	> 1.00
Betulinic acid	0.50	1.00	1.00
3-O-acetyllupeol	1.00	1.00	0.50
Lupeol	0.50	> 1.00	1.00
Myrianthic acid	0.50	1.00	1.00
Pomolic acid	1.00	> 1.00	0.50
Ursolic acid	1.00	1.00	0.50
Taraxerone	0.50	> 1.00	1.00
22,23-Dihydrospinasterol 3-O-β-D-glucopyranoside	> 1.00	> 1.00	1.00
Spinasterol	1.00	> 1.00	1.00
Spinasterol 3-O-β-D-glucopyranoside	> 1.00	> 1.00	1.00
Stigmasterol	> 1.00	> 1.00	1.00
Lacourtianamide	0.25	1.00	0.50
Lacourtianoside II	> 1.00	> 1.00	1.00
Epicatechin	0.25	0.50	0.50
Methyl pheophorbide-a	1.00	1.00	0.50

### Inhibition of biofilms by test compounds

The capacity of the compounds to inhibit biofilms in *S. aureus, E. coli* and *C. albicans* were evaluated at sub-MIC concentrations and percentage inhibitions reported on Table 5. It is always necessary to evaluate the effect of samples on planktonic bacterial and also in their biofilm forms at sub-MIC concentrations so as to have a proper reflection of their potential to eliminate the bacteria. All the compounds were able to inhibit bacterial biofilms in *S. aureus* at MIC and MIC/2 concentrations except 22,23-Dihydrospinasterol 3-O-β-D-glucopyranoside, Spinasterol, Spinasterol 3-O-β-D-glucopyranoside, Stigmasterol and Lacourtianoside II. At MIC/4 concentration, *S. aureus* biofilm formation was inhibited by Acetylerythrodiol (11.3 ± 0.4%), Betulinic acid (09.4 ± 0.7%), Myrianthic acid (22.8 ± 0.4%), Lacourtianamide (19.8 ± 0.9%), Epicatechin (28.4 ± 0.9%) and Methyl pheophorbide-a (14.6 ± 0.8%). At MIC/8, only Myrianthic acid (08.3 ± 0.2%), Lacourtianamide (07.0 ± 0.2%) and Epicatechin (11.7 ± 0.3%) were able to inhibit biofilm formation and these compounds exhibited the highest antibiofilm activity on *S. aureus*. The Gram-positive bacteria *E. coli* was the most difficult biofilm to inhibit as the percentage inhibitions were low and no inhibition was observed at MIC/4 and MIC/8. At MIC/2, only Erythrodiol (11.6 ± 0.8%), Betulinic acid (17.4 ± 0.5%), Lupeol (06.2 ± 0.1%), Ursolic acid (16.3 ± 0.2%), Lacourtianamide (05.7 ± 0.3%) and Epicatechin (25.5 ± 0.8%) were able to inhibit *E. coli* biofilm formation. Ursolic acid and Epicatechin had the highest biofilm inhibitions on *E. coli.* Against *C. albicans*, Erythrodiol, 22,23-Dihydrospinasterol 3-O-β-D-glucopyranoside, Spinasterol 3-O-β-D-glucopyranoside and Lacourtianoside II could not inhibit biofilm formation while at MIC/8 no biofilm inhibition was observed for the active compounds. At MIC/4, only Ursolic acid (08.8 ± 0.5%), Lacourtianamide (09.8 ± 0.4%) and Methyl pheophorbide-a (06.3 ± 0.1%) could inhibit biofilm formation in *C. albicans*. Ursolic acid, Lacourtianamide and Methyl pheophorbide-a had the highest biofilm inhibition percentages on *C. albicans* compared to the other compounds.

**Table 5:** Anti-biofilm activity of compounds

Compound	Microorganism								
	***S. aureus***				***E. coli***		***C. albicans***		
	**MIC**	**MIC/2**	**MIC/4**	**MIC/8**	**MIC**	**MIC/2**	**MIC**	**MIC/2**	**MIC/4**
Acetylerythrodiol	30.4 ± 0.6	19.9 ± 0.6	11.3 ± 0.4	-	06.9 ± 0.2	-	21.3 ± 0.5	08.6 ± 0.1	-
Amyrincapraote	39.2 ± 0.7	18.5 ± 0.8	-	-	-	-	24.1 ± 0.3	-	-
Erythrodiol 3-O-palmitate	34.2 ± 0.6	15.6 ± 0.2	-	-	-	-	29.0 ± 0.4	12.7 ± 0.1	-
Erythrodiol	36.2 ± 0.7	14.4 ± 0.4	-	-	27.1 ± 0.3	11.6 ± 0.8	-	-	-
3-O-acetylbetulin	18.4 ± 0.3	09.8 ± 0.4	-	-	16.3 ± 0.4	-	12.8 ± 0.6	-	-
Betulinic acid	37.3 ± 0.8	23.2 ± 0.6	09.4 ± 0.7	-	38.3 ± 0.9	17.4 ± 0.5	32.8 ± 0.4	14.9 ± 0.2	-
3-O-acetyllupeol	21.5 ± 0.8	06.6 ± 0.2	-	-	08.4 ± 0.3	-	21.4 ± 0.5	10.5 ± 0.2	-
Lupeol	29.6 ± 0.1	10.5 ± 0.7			23.7 ± 0.9	06.2 ± 0.1	16.4 ± 1.0	05.6 ± 0.2	
Myrianthic acid	62.4 ± 1.6	41.7 ± 0.5	22.8 ± 0.4	08.3 ± 0.2	08.9 ± 0.4	-	23.4 ± 0.3	07.85 ± 0.1	-
Pomolic acid	24.3 ± 0.3	07.6 ± 0.2	-	-	11.4 ± 0.2	-	27.7 ± 0.5	08.3 ± 0.1	-
Ursolic acid	38.7 ± 0.3	11.6 ± 0.5	-	-	34.3 ± 0.9	16.3 ± 0.2	47.5 ± 0.3	26.4 ± 0.4	08.8 ± 0.5
Taraxerone	27.9 ± 0.7	10.3 ± 0.4	-	-	14.9 ± 0.5	-	20.7 ± 0.4	04.4 ± 0.1	-
22,23-Dihydrospinasterol 3-O-β-D-glucopyranoside	-	-	-	-	-	-	-	-	-
Spinasterol	-	-	-	-	-	-	10.9 ± 0.5	-	-
Spinasterol 3-O-β-D-glucopyranoside	-	-	-	-	-	-	-	-	-
Stigmasterol	-	-	-	-	-	-	12.4 ± 0.7	-	-
Lacourtianamide	65.2 ± 1.4	41.7 ± 0.5	19.8 ± 0.9	07.0 ± 0.2	18.9 ± 0.6	05.7 ± 0.3	36.4 ± 0.6	16.5 ± 0.3	09.8 ± 0.4
Lacourtianoside II	-	-	-	-	-	-	-	-	-
Epicatechin	68.1 ± 2.3	45.7 ± 1.2	28.4 ± 0.9	11.7 ± 0.3	39.4 ± 0.9	25.5 ± 0.8	14.3 ± 0.4	-	-
Methyl pheophorbide-a	46.4 ± 0.9	25.7 ± 0.9	14.6 ± 0.8	-	-	-	38.3 ± 0.6	20.0 ± 0.5	06.3 ± 0.1

*-: No inhibition; values are means ± SEM for three parallel measurements*

## Discussion

Plant-derived compounds have different structural diversities, with different modes of action and are safer and cheaper than synthetic compounds, and this can be seen with the large number of discovered drugs from natural origin [28]. Various secondary metabolites from plants including phenolic compounds, alkaloids, β-lactam, macrolides, lectins, terpenoids, peptides and lipoglycopeptides, have been shown to possess antimicrobial activities with different modes of action such as inhibition of quorum sensing, efflux pump effects, biofilm inhibition and anti-motilities [29–32]. Violacein inhibition is easily measurable and reflects an anti-QS process in bacteria [33]. Violacein production contributes to the virulence factors in the *C. violaceum* CV12472 Gram-negative bacterium and its inhibition reflects the blocking of signal molecules that promotes communication within the bacterial colony. It can be seen from the results obtained that pentacyclic triterpenoids, the ceramide and epicatechin were able to inhibit violacein production in *C. violaceum* CV12472. Pentacyclic triterpenoids of the class of lupane and oleanane have demonstrated violacein inhibition and those containing the acid function were more potent, indicating that pentacyclic triterpenoids could be suitable scaffolds for the development of quorum quenching antimicrobials on various pathogens [34]. The violacein pigment production is mediated by QS system of genes dependent on CviR and it is helps microorganisms to coordinate processes such as population density and involves production and response to acylhomoserine lactones [35]. Since the bacteria *C. violaceum* produces violacein while growing, the inhibition of the production of this pigment reflects the inhibition of signal production. The mutant strain *C. violaceum* CV026 is unable to produce AHL and therefore can only produce violacein when an external AHL is supplied. The results in this study indicates that some compounds at certain concentrations were able to prevent *C. violaceum* CV026 from producing violaceum even when an AHL was supplied, and this reflects inhibition of signal reception by such compounds. QS inhibition can occur in multiple ways either by prevention of AHL signal production, disruption of AHL signal dissemination or through the interruption of AHL signal reception [36]. The reduction in the production of violacein pigment by *C. violaceum* CV12472 and the anti-QS zones against *C. violaceum* CV026 present as halos are visible on Fig. 3.

This is beneficial and can help to reduce bacterial virulence. QS communication systems help the bacteria to protect themselves from pressure exerted by antibiotic drugs and also to form self-protecting biofilm matrices on surfaces making treatments difficult [37–40]. Triterpenoids are able to impede various virulence factors including biofilm formation which increases microbial resistance to antibiotics and biofilms are regulated by QS through the inter-bacterial communication networks mediated by small signal molecules production and diffusion [41]. In one of such assays, triterpenoids of lupane and oleanane classes with carboxylic acid functional groups are very active and this can be justified as Betulinic acid, Myrianthic acid, Pomolic acid and Ursolic acid were amongst the active terpenoid compounds in all the assays. Equally, the most active compound could be considered to be epicatechin which was able to effectively inhibit QS and virulence factors with good inhibition of violacein pigment production, swarming motility and biofilm formation. Some plant extracts rich in epicatechin and other catechin derivatives have been shown to inhibit violacein, biofilms and swarming in bacteria [14].

Some pathogenic bacteria use coordinated flagella-driven movements called swarming on solid and semisolid surfaces to colonize surfaces, increase virulence and resistance antibiotics and this is a suitable way of bacteria to adapt to environmental challenges using signalling networks [42]. Most of the compounds were able to inhibit swarming motility against the model bacteria *P. aeruginosa* PA01. This ability to prevent swarming movement can be of interest for the development of new class of antimicrobials that can block coordinated behaviour in bacterial colonies including the movement towards nutrients, attachment and colonisation of surfaces and subsequent biofilm formation [43, 44]. It is beneficial to find new antimicrobial substances that can inhibit these QS regulated swarming motility which is a virulence factor exhibited by *P. aeruginosa* for adhesion unto surfaces and nutrient rich areas and to form biofilm [45]. Epicatechin showed the highest motility inhibition on *P. aeruginosa* PA01, and this confirms its ability to inhibit biofilms since swarming movement is involved in the early stages of biofilm formation. In one study, pure isolated catechin and an epicatechin-rich extract were able to inhibit pyocyanin pigment production as well as elastase synthesis and biofilm formation in *P. aeruginosa* PA01 thereby quenching QS-dependent virulence factors in this bacterium [46].

Swarming motility is also necessary for the dispersion of biofilms. Biofilms consist of extracellular matrix made up of self-produced substances, such as lipids, proteins and polysaccharides that protects the bacteria from disinfectants, antibiotics and host defence systems and contribute to increase resistance, and different antimicrobials that are capable of inhibiting biofilms and reducing virulence without killing the bacteria are important antimicrobial therapeutics [47, 48]. The ability of the test compounds to inhibit biofilm formation at low concentrations (sub-MIC), is a very good indication of their possible potential application to overcome microbial resistance developed by biofilms embedded pathogen cells. The results shown in this study therefore corroborates with previous studies that report inhibition of QS systems and biofilms by various triterpenoid scaffolds against many pathogens with promising potential [49, 50]. Various triterpenoids constitute bioactive phytochemicals that affects microbial biofilms and some structurally similar lupane and oleanane type triterpenoids with carboxylic acid groups substantially depleted biofilms to various extents, reducing surface and exo-polysaccharides of biofilm matrices [51]. It should be noted that bacteria within biofilms can’t be eliminated by ordinary antibiotics and these biofilm communities will continue to exercise pathogenicity and virulence even when planktonic communities are inhibited or killed. Biofilm-associated infections are chronic and are very difficult to treat with conventional antibiotics since most antibiotics must enter the cells meanwhile biofilms prevent antibiotics from entering bacterial cells. The development of non-biocidal strategies such as QS and biofilm inhibitions to combat bacterial infections is very crucial since it avoids antibiotic resistance contrary to the use of conventional antibiotics that can possibly lead to antimicrobial drug resistance [52].

## Conclusion

Conventional antibiotics which aim at killing bacteria or inhibiting their growth are usually challenged with antibiotic resistance and the fall out of use within short times of use. It is necessary to search for new antimicrobial substances which can overcome resistance and eliminate virulence factors and pathogenicity of bacteria. One important method to achieve this is to inhibit QS bacterial-communication systems. For this reason, twenty compounds isolated from the medicinal plant *G. lacourtiana* were evaluated for their inhibitory effects on QS and biofilm and the results indicated good potential especially for triterpenoids with carboxylic acid groups, the ceramide and epicatechin. Compounds from *G. lacourtiana* could serve as cheap starting materials for the development of antimicrobial drug which can possibly overcome microbial resistance since some of the compounds are capable of disrupting QS mediated processes in bacteria and biofilms.

## Data Availability

The data used to support the findings of this study are available from the corresponding author upon reasonable request.
